# A Wild *Fomes fomentarius* for Biomediation of One Pot Synthesis of Titanium Oxide and Silver Nanoparticles for Antibacterial and Anticancer Application

**DOI:** 10.3390/biom10040622

**Published:** 2020-04-17

**Authors:** Suriya Rehman, Romana Farooq, Rabindran Jermy, Sarah Mousa Asiri, Vijaya Ravinayagam, Reem Al Jindan, Zainab Alsalem, Manzoor A. Shah, Zafar Reshi, Hussein Sabit, Firdos Alam Khan

**Affiliations:** 1 Department of Epidemic Disease Research, Institute for Research & Medical Consultations (IRMC), Imam Abdulrahman Bin Faisal University, Dammam 31441, Saudi Arabia; 2 Department of Botany, University of Kashmir, Srinagar 190006, India; 3 Department of Nano-Medicine Research, Institute for Research & Medical Consultations (IRMC), Imam Abdulrahman Bin Faisal University, Dammam 31441, Saudi Arabia; 4 Department of Biophysics, Institute for Research & Medical Consultations (IRMC), Imam Abdulrahman Bin Faisal University, Dammam 31441, Saudi Arabia; 5 Deanship of Scientific Research and Department of Nano-Medicine Research, Institute for Research & Medical Consultations (IRMC), Imam Abdulrahman Bin Faisal University, P.O. Box 1982, Dammam 31441, Saudi Arabia; 6 Department of Microbiology, College of Medicine, Imam Abdulrahman Bin Faisal University, Dammam 31441, Saudi Arabia; 7 Department of Genetic Disease Research, Institute for Research & Medical Consultations (IRMC), Imam Abdulrahman Bin Faisal University, Dammam 31441, Saudi Arabia; 8 Department of Stem Cell Research, Institute for Research & Medical Consultations (IRMC), Imam Abdulrahman Bin Faisal University, Dammam 31441, Saudi Arabia

**Keywords:** antibacterial/anticancer, *Fomes fomentarius*, green synthesis, nanoparticles, silver/titanium

## Abstract

The present study offers an alternative method for green synthesis of the formation of two types of nanoparticles (NPs). These NPs, titanium oxide and silver NPs (TiO_2_ and Ag NPs, respectively), were obtained from the amalgamation of intracellular extract of a wild mushroom, *Fomes fomentarius*, with aqueous solutions of titanium isopropoxide and silver nitrate, respectively. *F. fomentarius* was identified phenotypically and by 18S ribosomal RNA gene sequencing (Gene accession no: MK635351). The biosynthesis of TiO_2_ and Ag NPs was studied and characterized by X-ray diffraction (XRD), diffuse reflectance UV-Visible spectroscopy (DR-UV), fourier transform infrared spectroscopy (FT-IR), scanning electron microscopy (SEM) and transmission electron microscope (TEM). Success was achieved in obtaining NPs of differing sizes and shapes. The antibacterial and anticancer activity of the NPs was significant with morphological damage being caused by both, although Ag NPs (10–20 nm) were found to have profound effects on bacterial and cancer cells in comparison to TiO_2_ NPs (100–120 nm). These metal NPs, synthesized using wild mushrooms, hold a great potential in biomedicinedue to an effective enzyme combination, which permits them to modify different chemical compounds to less toxic forms, which is required for ecofriendly and safe biomaterials.

## 1. Introduction

Nanoparticles (NPs) possess rare chemical, optical, photoelectrochemical, magnetic and catalytic characteristics, having potential in medicine and other disciplines [1]. Among the vast array of NPs, Ag NPs are the most stable and biocompatible due to their unusual physical and chemical properties. Ag NPs are also known to possess antibacterial, antifungal, antiviral, antioxidant, anti-inflammatory and antitumor activities, and hence they have broad possibilities as use as nano-Ag in biomedicine for antimicrobial drugs, medical devices, medicinal products and cosmetics [2,3,4]. Inert and nontoxic TiO_2_ is another economical material that is highly capable of absorbing UV light, due to its high refractive index. TiO_2_ NPs are extensively used in cosmetics, paper, paints, food colorants, inks and toothpaste, due to their white pigment and the fact that they are environmentally friendly catalysts [5]. Various physicochemical and biological properties, as well as the toxicity of NPs, are because of the shape and size of NPs [6,7,8]. The control of NP morphology with desired characteristics demands new measures in techniques of synthesis involving chemical and green synthesis routes. Recently, AgNPs (~15 nm) were reported using chemical methods through an ultrasonic assisted route for metal-based conductive ink technology. The chemical method used ascorbic acid and glucose as a mixed reducing agent in poly(N-vinylpyrrolidone) solution [9]. Nano TiO_2_ with major anatase phase (>94%) has been reported using a chemical vapor condensation technique. The experiments involve the introduction of a titanium source (Titanium tetraisopropoxide), argon and air in the vapor phase into an alumina tube for subsequent thermal decomposition to produce TiO NPs [10]. Nanoparticle preparation through green synthesis is the emerging field of research that provides an alternative route to conventional chemical methods that use tedious experimental set-up and eco-unfriendly chemicals. Present times have identified an increased adoption of eco-friendly approaches for the synthesis of NPs, such as the use of plants, bacteria, fungi and algae, etc. [11,12,13,14,15]. AgNPs and starch nanoparticles has been reported using cheap, biodegradable resources such as pomegranate seed (*Punica granatum* L.) and starch for food packing film application. The study shows that sunlight can be effectively used as a photo-reducing agent to convert silver nitrate sources to AgNPs [16]. Biosynthesis of AgNPs with a particle size of about 15 nm was reported using two white rot fungal strains (*Ganoderma enigmaticum and Trametes ljubarskyi*). The nanoparticle synthesis via the extracellular biosynthetic route using organism extract as reducing agents was found to be economically viable with high growth rates in lab scales [17]. Green synthesis of TiO_2_ NPs was reported using *Azadirachta indica* leaf extract. The presence of active components (terpenoids, flavonoids and proteins) was shown to stabilize the crystalline nanoparticle formation in sizes ranging between 15–50 nm [18].

In this regard, the use of fungi for nanoparticle synthesis, particularly the unexplored higher fungi (mushroom) is still in its infancy. The most familiar species of mushrooms belong to the group *Basidiomycota*, polyporales, which constitutes an order of about 1800 species of fungi in the division [19]. Polypore, a term used for basidiocarp-producing fungi, appears tough and leathery, typically large (>3 cm), and found mostly on live and dead trees [20]. These basidiomycetes are nonpathogenic, nontoxic and can be grown in pure cultures, hence, they are favorable for the biosynthesis of NPs [21]. The use of basidiomycetes for NP synthesis has not been extensively explored, as compared to synthesis using lower fungi and bacteria [22]. Herein, we report the mediation of culture filtrate of wild basidiomycetes, *F. fomentarius* for the green synthesis of TiO_2_ and AgNPs and subsequent in vitro studies for antibacterial and anticancer activity.

## 2. Experimental

### 2.1. Collection, Phenotypic and Genotypic Studies of F. fomentarius

For the collection of sporocarps, a standard method was followed [20]. Photographs were taken by a Nikon D5300 DSLR Camera (Nikon, Tokyo, Japan) with a zoom lens of 18–140 VR (data of sampling in supplementary file). Passport data and the microhabitat characteristics of collected samples has been recorded in the field book. Samples were properly labeled, given a voucher number and carried to the laboratory for detailed morphometric examination.

Collected specimens were identified by keen observation of structures like pileus, stipe, their shape, structure, gill attachment, etc., using standard keys (e.g., Mycokey, Index fungoram etc.) field guides and manuals. The samples were dried and deposited at the herbarium of the Centre for Biodiversity and Taxonomy, University of Kashmir, J&K, India. Microscopic features and measurements were made from slides that were prepared and stained with lactophenol cotton Blue, 2% KOH and Melzer’s reagent. For examination, the spores were tapped off the razor blade onto a clean and a drop of KOH or Melzer’s reagent was added. Observation and photographs were captured at magnification between ×40 to ×100 using a Nikon Eclipse 80i microscope and phase contrast illumination (Nikon, Tokyo, Japan).

#### 2.1.1. DNA Isolation and PCR

DNA extraction was done using the manual CTAB method (cetyl trimethylammonium bromide) [23]. The extracted DNA was dissolved and preserved in TE (Tris-EDTA) buffer. The amplification was carried out for internal transcribed spacer (ITS) regions using the ITS1 and ITS4 in a PCR System Thermocycler Applied Biosystems with following parameters—10 min of initial denaturation at 95 °C, 35 cycles at 95 °C for 1 min, 54 °C for 30 s and 72 °C for 2 min, followed by extension at 72 °C for 10 min. The purification of amplified products was done and sequenced with the same primers. The DNA sequences were submitted to GeneBank and analyzed for homology using BLAST on NCBI [24] (Table 1).

#### 2.1.2. Sequence and Phylogeny Analysis

Wild mushroom was identified by ribosomal gene analysis. The small subunit sequences were aligned with additional sequences downloaded from NCBI GenBank (http//ncbi.nim.nih.gov) using BioEdit Sequence Alignment Editor (version 7.2.5) [25]. The sequence alignments and phylogenetic analysis were performed using MEGA 10 software (Tamura et al., 2011). Phylogeny was studied on ITS -18SrRNA genes by maximum likelihood method. Initial alignment was done using Clustal W software for maximum alignment and minimum gaps. The tree was generated by using the program DNADIST and NEIGHBOR from PHYLIP 3.69 [24].

### 2.2. Biosynthesis of TiO_2_ and AgNPs Using Fomes Fomentarius

The synthesis of TiO_2_ and AgNPs was conducted using the extract of *F. fomentarius* by adopting a green synthesis method [23]. The *F. fomentarius* sample was dried to obtain powder (10 g), which was further mixed with 100 mL of millipore water and sonicated for 25–30 min. The mixture was further centrifuged at 4000 rpm to obtain the clarified solution. Subsequently, solution was filtered and stored at 4 °C. A total of 10 mL of filtrate was mixed with 1 mM AgNO_3_ (100 mL) and put at room temperature on a shaker for agitation under observation, until the appearance of color change (10 min). A similar procedure was followed for TiO_2_ NPs, where 100 mL of 1 mM Titanium (IV) isopropoxide was used as a source solution.

### 2.3. Characterization of Biosynthesized TiO_2_ and Ag NPs

The crystalline phase of TiO_2_ and Ag NPs was measured using a benchtop X-ray powder diffractometer MiniFlex 600 (Rigaku, Shibuya, Tokyo, Japan). The sample was measured in 2 theta range 5–80°, with step size of 0.02° and scan rate of 1°/min. The coordination environment of TiO_2_ and Ag NPs were analyzed using diffuse reflectance UV-Visible spectroscopy (V-750, JASCO). The sample for diffuse reflectance was prepared by dispersing the sample in a spherical disc with an integrated sphere (60 mm dia, ISV-922). After pressing, the sample with 0.5 mm thickness was scanned between wavelength range 200–870 nm. The TiO_2_ and Ag NPs functional groups were analyzed using fourier transform infrared spectroscopy equipped with attenuated total reflectance (ATR) (Perkin Elmer, Arcata, CA, USA). The surface morphology, distribution and features of TiO_2_ and Ag NPs were studied using scanning electron microscopy (SEM) (Inspect S50) and transmission electron microscope (TEM) (Morgagni 268). For TEM analysis, samples were prepared by dispersing in ethanol followed by shaking in an ultrasonicator for 20 min, and then a suspended drop was dried at room temperature on the carbon-coated copper grid [15].

### 2.4. Antibacterial Activity of Biosynthesized NPs

Common pathogenic bacteria *Escherichia coli* (*E. coli* ATCC35218) and *Staphylococcus aureus* (*S. aureus* ATCC29213) were used for the antibacterial activity of synthesized TiO_2_ and Ag NPs by agar well diffusion. The bacterial strains were maintained on nutrient agar media (NA). In preparation for the antibacterial study, a homogeneous water suspension of the NPs was prepared by sonication for 15–20 min at 30 °C. Test organisms grown at 37 °C for 18 h in Mueller Hinton (MHB) were adjusted to the cell density of 10^6^ CFU/mL. A total of 100 µL of adjusted inoculum of each bacterial strain was inoculated on the MHA plates. After 20–30 min, the dried plates were punched for wells using the sterile borer. A total of 50 µL of TiO_2_ NP and Ag NP (100 µg/mL) suspension was placed into the wells. Sterile water was used as a negative control. This was followed by the incubation at 37 °C for 24 h. The activity of the synthesized NPs was evaluated by measuring the zone of inhibition zone around the wells in millimeters (mm) [15].

### 2.5. Study of Topological Changes in Treated Bacteria by SEM

Additionally, the treated *E. coli* and *S. aureus* were studied by SEM for the morphological and physiological alteration caused by NPs. Precisely, adjusted bacterial cells were treated with 100 µg/mL of TiO_2_ and Ag NPs and further incubated at 37 °C for overnight. Later, the incubated mixture was centrifuged at 12,000 rpm for 10 min for treated and untreated cells. The harvested cells were thrice washed using PBS and primarily fixed with 2.5% glutaraldehyde for 4 h, followed by fixation with 1% osmium tetroxide for 2 h. Cells were washed multiple times and further dehydrated by varying concentrations of ethanol (50%, 70%, 90%, 100%). The cells were placed onto the aluminum stubs and dried using a desecrator. Finally, gold coating was done and cells were examined by SEM at an accelerating voltage of 20 kV [26].

### 2.6. Cytotoxic Activity

#### 2.6.1. Cell Culture & Treatments

Human colorectal carcinoma cells (HCT-116) were used for the study. DMEM medium was used, which was supplemented with 10% fetal bovine serum (FBS); (10%) L-glutamine; 10% selenium chloride; 120 μg/mL and streptomycin; and 120 Unit/mL penicillin in a 5% CO_2_ incubator (Thermo Scientific Heracell-150, Langenselbold Germany) at a temperature of 37 °C. The cells with more than 70–80% confluency were used for the TiO_2_ and Ag NPs treatments. The treatment of HCT-116 cells was carried out with different concentrations of NPs ranging from 0.5 to 8.0 µg/mL. The cells were analyzed after a time span of 48 h. The experiment was carried out in triplicate for statistical analysis [27].

#### 2.6.2. Cancer Cell Morphology

The cell morphology of untreated and treated HCT-116 cells was examined post-48 h under an inverted microscope (TS100F-Eclipse, Nikon, Tokya, Japan) and compared under 200× magnification.

#### 2.6.3. Cytotoxicity by MTT Assay

The cells with confluency of 70–80% in 96-well cell culture plates were subjected to MTT assay. After 48 h, MTT (5 mg/mL) was added in all the wells and kept for 4 h. Later, DMSO was added and the plate was read in an ELISA Plate Reader 570 nm wavelength (Biotek Instruments, Winooski, VT, USA). The (%) percentage of cell viability was calculated as per given formula:

Cell Viabilty % = (A/B) × 100(1)

A: optical density of nanoparticles, B: optical density of controls.

#### 2.6.4. Nuclear Staining by DAPI

The cells were stained with DAPI staining to study the effect of TiO_2_ NPs and Ag NPs on the cell nucleus. After 48 h, the treated and untreated HCT-116 cells were immersed in ice-cold (4%) paraformaldehyde. Later, the cells were added with Triton X-100 and prepared in PBS for 5 min to premetallize the cell membrane. The cells were stained using DAPI (5 μg/mL) in PBS, prepared in dark. Washing with Triton X-100 was done, followed by examining the nuclear morphology under confocal scanning microscope (Zeiss, Jena, Germany) equipped with a digital camera [27].

## 3. Results and Discussion

### 3.1. Phenotypic and Genotypic Studies of F. fomentarius

Various wild mushrooms are recorded to have potential anticancer and antioxidant properties, specifically, edible mushrooms possess several bioactive molecules with unique and diverse bioactivities, like antimicrobial, anti-inflammatory, antioxidant, antitumor and anticancer activities [28]. Based on these reports, an attempt was made to use *F. fomentarius* extracts as reducing agents for the synthesis of TiO_2_ and AgNPs, which was collected from an angiosperm host in the natural forest of Kashmir valley, India. This wild mushroom has been recently reported to possess anti-inflammatory, antioxidant, antinociceptive, antidiabetic, antibacterial, and cytotoxic activities.

**Figure 1:**
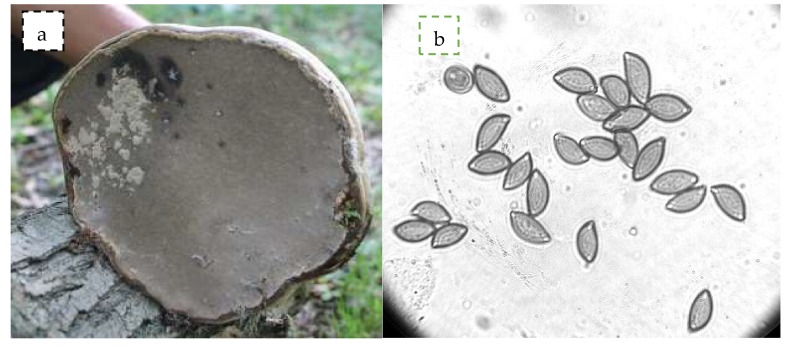
Photographs of brown rot basidiomycetes—*F. fomentarius*, (**a**) under view showing pores (**b**) basidiospores at 100×.

The upper side of *F. fomentarius* is zoned concentrically with wavy furrows. The basidiomes are perennial, leathery and hoof-shaped. The above surface is smooth and zoned, having a thick crust, and the lower surface is pale brown and concave in shape (Figure 1a). The microscopic observations were mainly focused on basidiospores, which were cylindrical to ellipsoid in shape, measuring 36 × 1.5 to 2 µm. Spores are bilaterally asymmetrical (inequilateral), as they are forcibly discharged from the basidium for dispersal. The shape of the hilar appendix is beaked. The spore apex is rounded. Spore ornamentation is smooth (Figure 1b).

The ITS1-ITS4 sequences of *F. fomentarius* were deposited in the NCBI Gene Bank under accession number MK635351. The phylogenetic relationships with related species are shown in Table 1 (phylogenetic tree is included as supplementary information).

### 3.2. Characterization of TiO_2_ and AgNPs

**Figure 2:**
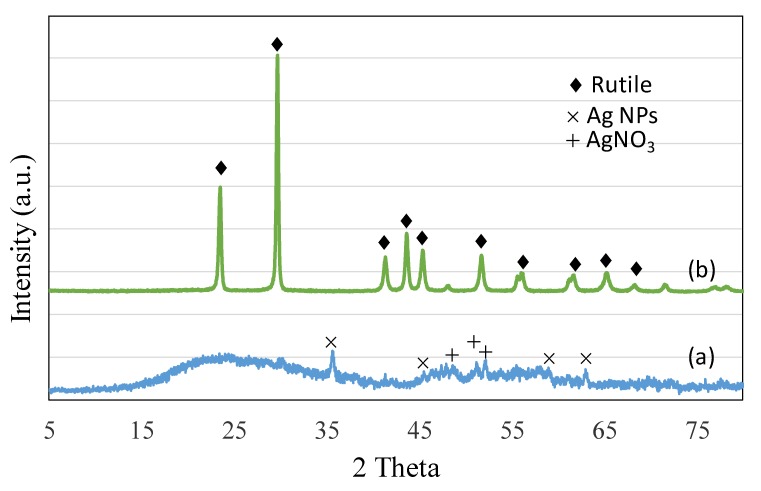
Powder X-ray diffraction spectra of (**a**) Ag nanoparticles (NPs) and (**b**) TiO_2_ NPs. The spectra (**a**) represents Ag NPs with FCC structure and (**b**) shows TiO_2_ NPs crystals with rutile phase

Figure 2a,b shows the X-ray diffraction (XRD) spectra of Ag and TiO_2_ NPs. In the case of Ag NPs, clear diffraction lines corresponding to (111), (200) and (220) planes were observed, indicating the presence of face-centered cubic (fcc) crystals. The presence of an additional peak at a 2-theta value of about 51.2 and 52.3 and additional less indexed peaks can be ascribed due to AgNO_3_ compounds present in the extract. Khatami et al. (2018) found a similar additional peak and ascribed it to the additional compounds present in the dried grass extract [29]. In the case of TiO_2_ NPs, the formation of crystalline TiO_2_ was observed with sharp peaks corresponding to the rutile phase.

**Figure 3:**
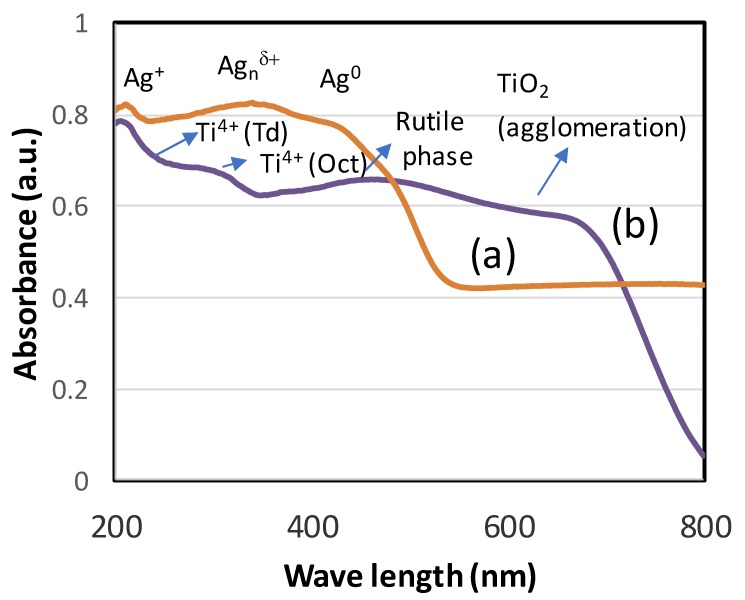
Diffuse reflectance UV-visible spectra of (**a**) Ag NPs and (**b**) TiO_2_ NPs. The spectra (**a**) shows existence of Ag+, Ag_n_^δ+^ and Ag^0^ species and (**b**) shows isolated Ti(IV), Octahedral Ti and rutile phase of TiO_2_ NPs.

Diffuse reflectance UV-visible spectra were recorded to study the coordination site of titanium oxide and Ag NPs in the extract. Figure 3a,b shows the diffuse reflectance spectra of Ag NPs and TiO_2_ NPs. The synthesized silver nanoparticle showed the presence of different oxidation states of Ag species (Figure 3a). The presence of three clear bands was observed at 220, 350 and 410 nm. The small band at 220 nm was ascribed due to Ag^+^, while a broad band at 350 and 410 nm showed the dominant species of Ag_n_^δ+^ nanoclusters and Ag^0^ species. In the case of TiO_2_, the band at 220 nm shows the presence of isolated Ti(IV) species, while the octahedral Ti species was found at about 300 nm. In line with XRD analysis, the sample TiO_2_ showed the presence of a rutile (titania) phase at about 410 nm and expanded to show the presence of agglomeration among TiO_2_ nanoparticles with broad absorption extending up to 700 nm.

**Figure 4:**
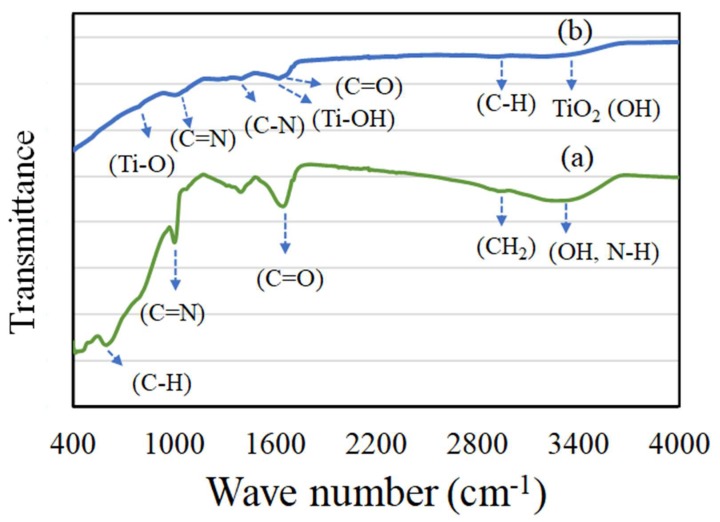
Fourier transform infrared (FT-IR) spectra of (**a**) Ag NPs and (**b**) TiO_2_ NPs. The spectra (**a**,**b**) shows the presence of various phytocomponents related to amino, methyl and hydroxyl groups assist in Ag and TiO_2_ NPs formation.

Figure 4a,b shows the Fourier transform infrared (FT-IR) spectroscopy of Ag and TiO_2_ NPs. The presence of active components like flavonoids and alkaloids in the extract are reported to play an active role in reducing Ag^+^ ions of a metal source to Ag NPs. A reduction in peak intensity and peak position compared to the extract indicates an effective nanoparticle formation [30]. In the case of mushroom extract, generally an intense peak appears corresponding to the presence of an amino and hydroxyl functional group [31]. In our case, Ag NPs exhibited a broad peak at about 3290 cm^−1^ corresponding to hydroxyl (-OH) and N-H stretching of primary amines (Figure 4a). A methylene C-H stretching peak was observed at 2940 cm^−1^. The presence of an asymmetrical C-O stretching peak was observed at 1655 cm^−1^. The presence of aromatic and aliphatic amines (C-N) was clearly seen with an intense absorption peak at about 1406 and 1000 cm^−1^. In the case of TiO_2_ NPs, comparatively less intense peak absorption values were observed between 3000–3680 cm^−1^ corresponding to -C-H symmetric stretching (2956 cm^−1^) and the hydroxyl group of TiO_2_ (3420 cm^−1^). The presence of a hydroxyl band Ti-OH was clearly observed at 1630 cm^−1^. Further, the presence of TiO_2_ NPs was confirmed with absorption peaks between 766–1630 cm^−1^, corresponding to Ti-O, aliphatic C-N, and aromatic C=N bands. The study showed the presence of various phytocomponents related to amino, methyl and hydroxyl groups present in the mushroom sample assist in transformation into silver and titanium oxide NPs.

**Figure 5:**
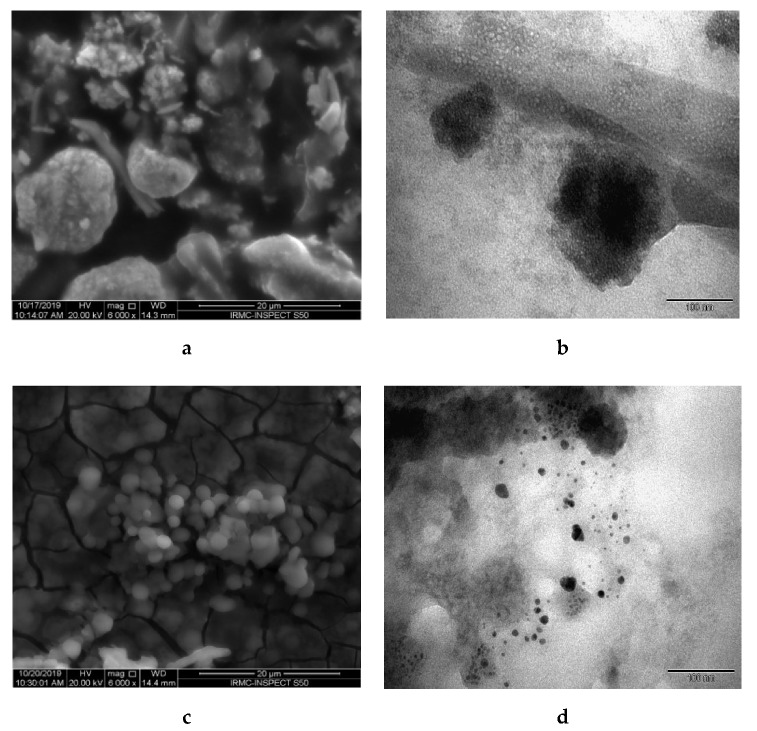
Morphological study of Ag and TiO_2_ NPs—(**a**) scanning electron microscopy (SEM) and (**b**) transmission electron microscope (TEM) images of TiO_2_ NPs; (**c**) SEM and (**d**) TEM images of Ag NPs.

Figure 5 depicts SEM and TEM morphology of TiO_2_ and Ag NPs. Figure 5a shows that TiO_2_ NPs were uniformly distributed on the surface with irregular shape and formation of aggregated NPs. The observed micrograph shows aggregates of TiO_2_ NPs with a rough surface. Meanwhile, the TEM micrograph of TiO_2_ corresponded with SEM results, which showed that the prepared NPs are asymmetrical particles with an average diameter of around 80–120 nm. For Ag NPs, SEM morphology (Figure 5c) illustrated an almost spherical shape with smooth surface conglomerated with each other [32]. TEM showed small spherical NPs distributed with an average diameter of around 10–20 nm.

Previous studies have found mushroom species, which mediate the formation of NPs, contain adequate proteins and enzymes and are an important group of fungi, with medicinal properties [33]. However, the exact mechanism involved in the conversion of NPs via mushroom extract is still unclear and needs a detailed study.

### 3.3. Antibacterial Activity of Synthesized NPs

**Figure 6:**
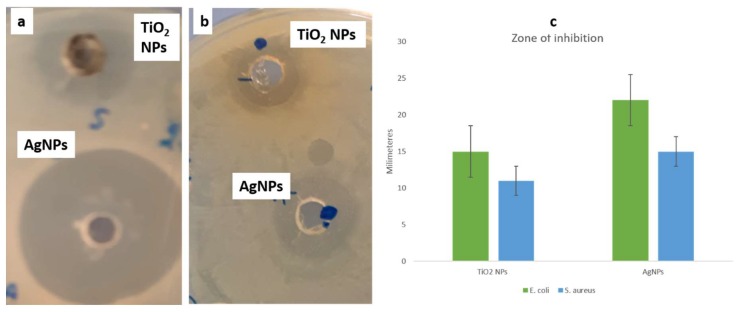
Zone of inhibition by agar well diffusion at 100 µg/mL of NPs; (**a**) *E. coli* and (**b**) *S. aureus* (**c**) Graph depicting ZOI.

The antibacterial activity of TiO_2_ NPs and Ag NPs was performed by an Agar well diffusion method using *E. coli and S. aureus*. The zone of inhibition was seen for both the gram-positive and gram-negative species against both the tested NPs (Figure 6). *E. coli* had a clear zone of 15 mm and 22 mm in diameter, against TiO_2_ NPs and Ag NPs, respectively. Whereas *S. aureus* was observed with 11 and 15 mm of clear zones, against TiO_2_ NPs and Ag NPs, respectively. The obtained results indicated that both the NPs have significant activity against both bacteria, with the elevated activity obtained against *E. coli*, when treated with Ag NPs.

This study of antibacterial activity of TiO_2_ NPs and Ag NPs against the Gram-negative and Gram-positive bacteria depicted that NPs could arrest the functioning of the cell. Ag NPs are more effective obstructing agents, as silver ions (Ag^+^) get released from Ag NPs and interact with the phosphorus moieties in bacterial DNA, leading to inactivation of replication, therefore preventing growth [33]. The current results agree with several studies conducted previously, pointing towards the antibacterial activity of biosynthesized Ag NPs using mushrooms, however, this is a first report of synthesis of mushroom-mediated TiO_2_ NPs, to the best of our knowledge.

Nithya and Ragunathan synthesized NPs by *Pleurotus sajor-caju*, which was studied against *P. mirabilis* and *P. Aeruginosa*, and recorded the zone of inhibition of 14 and 12 mm, respectively [34]. Manzoor et al. studied Ag NPs through *Agaricus bisporus*, which is a nutritionally and medicinally important species of mushrooms [31]. Birla et al. recorded enhanced activity of *E. coli* and *P. aeruginosa* compared to *Staph aureus by Ag NPs synthesized through* *Phoma glomerata* [35]. Additionally, Panáček et al. and Balaji et al. suggested that Ag NPs could be combined with antibiotics for better efficacy against number of pathogenic microbes [36,37].

The present study is also in agreement with the studies conducted by Swathi et al. on TiO_2_ NPs synthesized by the green method, against Gram-negative and Gram-positive bacteria, indicating the elevated activity against Gram negative organism [23,38].

**Figure 7:**
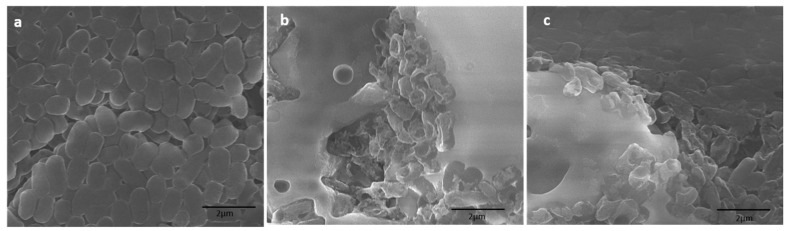
SEM micrographs of *E. coli:* (**a**) control (untreated cells); (**b**) Ag NPs at 100 µg/mL; (**c**) TiO_2_ NPs at 100 µg/mL.

Topological changes caused by synthesized NPs in *E. coli and S. aureus* were further evaluated by SEM. The untreated (control) *E. coli* cells appeared to be rod-shaped, having a consistent and intact cell surface (Figure 7a). However, treated *E. coli* cells were no longer intact, with abnormal and irregular appearance at the cellular surfaces (Figure 7b). The cells treated with Ag NPs appeared more affected than those of the TiO_2_ NPs (Figure 7c). The *E. coli* cells treated with TiO_2_ NPs showed mild alteration, whereas *E. coli* cells were severely damaged by Ag NPs. This was due to pit formation and distortion of cellular wall and membrane, reflecting the loss of the cellular integrity, which possibly cause the bacterial death.

**Figure 8:**
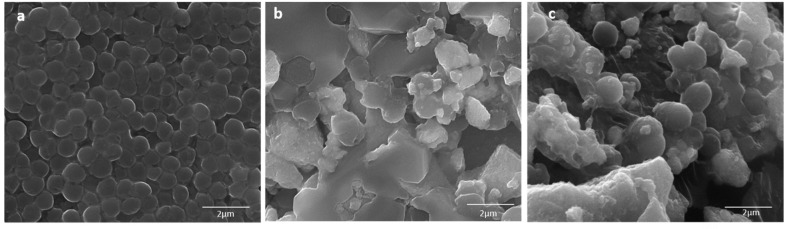
SEM micrographs of *S. aureus*: (**a**) control (untreated cells); (**b**) Ag NPs at 100 µg/mL; (**c**) TiO_2_NPs at 100 µg/mL.

On the other hand, the control cells (untreated) *S. aureus* cells were found in normal coccus shape, with a smooth and continuous cell surface (Figure 8a). Contrary to this, the treated *S. aureus* cells were irregular in shape and had a distorted cell surface. Both the samples, Ag NPs and TiO_2_ NPs, had almost similar effects on Gram-positive cells. The cell surface was seen as irregular with a distorted cellular surface. The obtained results suggested that the *E. coli* cells were more severally affected as compared to *S. aureus*, when treated with Ag NPs (Figure 8b,c).

The obtained results demonstrated the significant activity with Ag NPs, which could be owing to their smaller size of 10–20 nm in comparison to TiO_2_NPs, ranging in size from 80–120 nm. The enhanced activity could be also due to the efficient attachment of spherical-shaped Ag NPs to the cellular surface, which could play a vital role in achieving good bactericidal activity. Although there are several reports about the antimicrobial action of silver nanoparticles, the actual mode of action is still unclear [39,40]. Some studies speculate the interaction of physical entities and electrostatic forces between the positively charged NPs and the negative charge on the cell surface of bacteria [41,42]. The potential of Ag NPs as antimicrobials can be credited to various possible metabolic processes, like inactivation of enzymes and proteins, degradation of DNA, etc. [35]. The future prospectus of these NPs lies in their relatively smaller size and increased surface area, which might have a huge impact on metabolic processes like respiration, energy generation and permeability to pathogens [41]. Ag NPs are reported to easily attack proteins which contain phosphorous and sulfur as the cell constituent and genetic material, leading to cell lysis [3,7]. Such properties of Ag NPs have potential for the development of effective antimicrobial drugs for application in food packaging materials and other durable polymeric materials.

Quite recently, the antibacterial activity of green-synthesized TiO_2_ NPs has been reported against two pathogens, *E. coli* and *S. aureus* [38]. Lusvardi et al. demonstrated the formation of spherical aggregates of TiO_2_ NPs, having a profound activity in the reduction of colony count of a bacterial strain *Pseudomonas* [43]. From the above results, it becomes clear that the synthesized NPs by a green approach is not only environmentally friendly, but also has a great future in pharmaceutical and biomedical industries.

### 3.4. Anticancer Activity of Synthesized NPs

**Figure 9:**
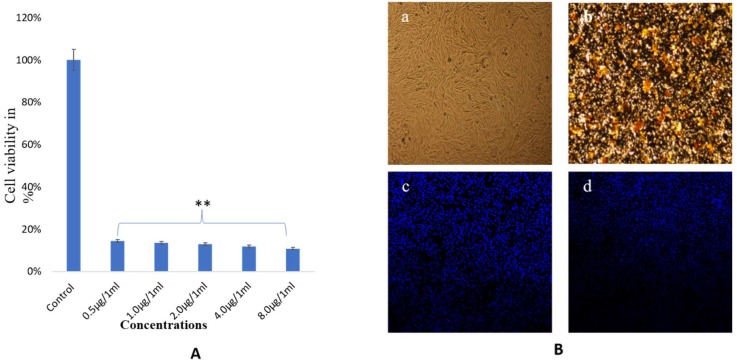
Cell viability by MTT Assay. The HCT-116 cells treated with different concentrations of Ag NPs after 48 h (**A**). (**B**). Cell morphology of HCT-116 cells on treatment with (**a**) Ag NPs control and (**b**) treated with 8.0 μg/mL, analyzed by a light microscope. (**c**) Control and (**d**) treated with 8.0 μg/mL analyzed by a confocal scanning microscope. Difference between two treatment groups were analysed by student’s t test where ** *p* < 0.01.

The impact of biosynthesized NPs was examined for microscopic observations and by an MTT assay. Both TiO_2_ and Ag NPs showed dose-dependent effects on cancer cell survivability, as examined by MTT assay. The treatment of AgNP_3_ also showed strong cytotoxic effects on cancer cell viability, as a larger majority of the cells were found dead after treatments of lower than 0.5 μg/mL (Figure 9A). The treatment exhibited significant alterations in cell morphology and the cell nucleus, as revealed by DAPI staining. Clear evidence of condensation and disintegration of the nucleus was seen, with lots of cancer cells found dead during the observation. NP treatment caused significant loss of nuclear staining as compared to control cells (Figure 9B). Data represented are the means ± SD of three replicated experiments. No significant damage was found in the control group.

**Figure 10:**
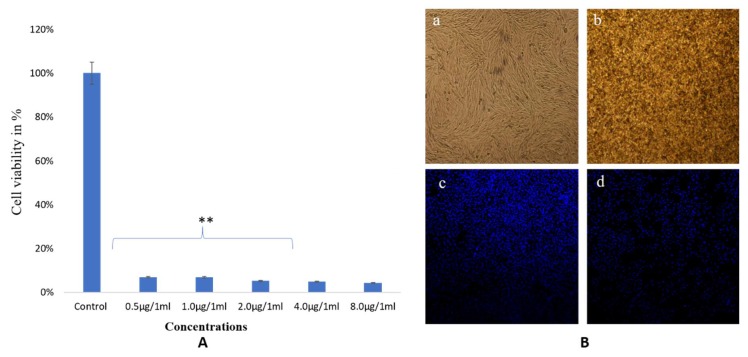
Cell viability by MTT Assay. The HCT-116 cells treated with different concentrations of TiO_2_ NPs after 48 h (**A**). Cell morphology of HCT-116 cells on treatment with TiO_2_NPs (**B**). (**a**) control and (**b**) treated with 8.0 μg/mL, analyzed by a light microscope. (**c**) Control and (**d**) treated 8.0 μg/mL analyzed by a confocal scanning microscope. Difference between two treatment groups were analysed by student’s t test where ** *p* < 0.01.

The treatment with TiO_2_ NPs also showed strong cytotoxic effects on cancer cell viability as a larger majority of the cells were found dead after treatments of lower than 0.5 μg/mL (Figure 10A). Significant changes in cell structure and nucleus were depicted by DAPI staining. The nuclear disintegration and condensation were indicated, with many dead cells seen. Control cells were found unaffected during treatment (Figure 10B).

Ag NPs have been reported to activate the apoptotic pathway via generation of free oxygen radicals, which result in antitumor and antiproliferative effects. Such nanomaterials that have antiangiogenic activities are known for promising abilities to alter the mechanism of proteins that are expressed abnormally [44,45]. Additionally, TiO_2_ NPs synthesized via green methods have also been reported to possess antiproliferative activity against cell lines such as the Mg 63 osteosarcoma and rat embryo fibroblast lines [46,47]. However, the exact mechanism of cancer cell death at the molecular level is still to be fully known. Therefore, it would be fascinating to unravel the apoptotic pathways involved in TiO_2_ and Ag NP-mediated cancer cell death. Interestingly, there are several reports of NPs that are known to cause nuclear fragmentation and disintegration in various cancer cell lines [48].

## 4. Conclusions

The aqueous extract of *F. fomentarius* from Kashmir Himalaya mediated the synthesis of TiO_2_ and Ag NPs with varying sizes and shapes. The chemical environment of NPs and morphological features were characterized using different characterization tools. The antibacterial and anticancer activity depicted the significant effect of NPs on the tested cells. Hence, the present study supports the green synthesis of TiO_2_ and Ag NPs, using a wild mushroom, as an environmentally sustainable approach.

## Figures and Tables

**Table 1:** Gene Bank accession numbers and top BLAST match sequences of the mushroom isolates along with maximum identity and query coverage.

Accession Number	BLAST Match Sequence		
	Reference Accession Number	Coverage	Maximum Identity
MK635351	JX126894.1 *Fomes fomentarius*	100%	100%
	KU1391991.1 *Fomes fomentarius*	100%	100%
	MK9101131 *Fomes fomentarius*	100%	99.82%
	KU863082.1 *Fomes fomentarius*	100%	99.82%
	KX065943.1 *Fomes fomentarius*	100%	99.82%

## Supplementary Materials

The following are available online at https://www.mdpi.com/2218-273X/10/4/622/s1, Figure S1: Phylogenetic relationship of *Fomes fomentarius* (MK635351) ITS sequencnces with other related members based on maximum likelihood method inferred from ITS sequences. Table S1: STUDY AREA.
