# Supplementary material for: A Wild Fomes fomentarius for Biomediation of One Pot Synthesis of Titanium Oxide and Silver Nanoparticles for Antibacterial and Anticancer Application
Source: Biomolecules. 2020 Apr 17;10(4):622. doi: 10.3390/biom10040622 (PMC7226099; doi:10.3390/biom10040622)
Supplement: Supplementary file 1 [file biomolecules-10-00622-s001.pdf]

A wild *Fomes fomentarius* for Biomediation of One pot Synthesis of Titanium Oxide and Silver Nanoparticles for Antimicrobial and Anticancer *Application*

SAMPLING:

Extensive field surveys were conducted in the coniferous forests of Budgam, Ganderbal, Anantnag, Baramullah and Pulwama district of Kashmir valley during 2016-2018 growing seasons of mushrooms.

SITE NAME	ALTITUDE MASL	LATITUDE	LONGITUDE	SITE CHARACTERISTICS	DISTRICT
Mammer	2400 m	34°14′N	75°01′E	Open forest areas with coniferous trees	Ganderbal
Kellar	1630 m	33 °46′N	74°46′E	Open coniferous forests	Pulwama
Gulmarg	2703m	34°03′N	74°23′E	Dense forest area with mixed plantation	Baramulla
Yusmarg	2400m	33°50′N	74°38′E	Open forests some grassy fields	Budgam
Pahalgam	2740 m	34°01N	74°31′E	Dense Coniferous forests	Anantnag

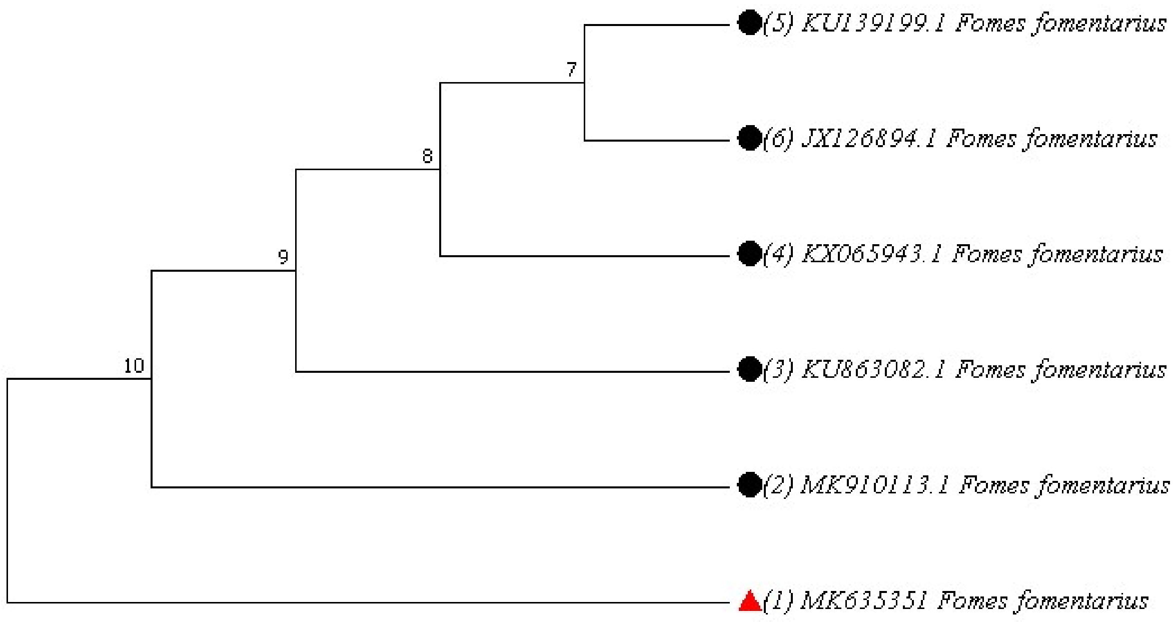

Figure: Phylogenetic relationship of *Fomes fomentarius* (MK635351) ITS sequencnces with other related members based on maximum likelihood method inferred from ITS sequences.
